# Global Epidemiology of Tuberculosis and Progress Toward Achieving Global Targets — 2017

**DOI:** 10.15585/mmwr.mm6811a3

**Published:** 2019-03-22

**Authors:** Adam MacNeil, Philippe Glaziou, Charalambos Sismanidis, Susan Maloney, Katherine Floyd

**Affiliations:** ^1^Division of Global HIV and TB, Center for Global Health, CDC; ^2^Tuberculosis Monitoring and Evaluation, Global Tuberculosis Programme, World Health Organization, Geneva, Switzerland.

Worldwide, tuberculosis (TB) is the leading cause of death from a single infectious disease agent (*1*) and the leading cause of death among persons living with human immunodeficiency virus (HIV) infection, accounting for approximately 40% of deaths in this population (*2*). The United Nations’ (UN) Sustainable Development Goals (*3*) and the World Health Organization’s (WHO’s) End TB Strategy (*4*) have defined ambitious targets for 2020–2035, including a 35% reduction in the absolute number of TB deaths and a 20% reduction in TB incidence by 2020, compared with 2015 (*4*). Since 2000, WHO has produced annual TB estimates for all countries (*1*). Global and regional disease estimates were evaluated for 2017 to determine progress toward meeting targets. In 2017, an estimated 10 million incident cases of TB and 1.57 million TB deaths occurred, representing 1.8% and 3.9% declines, respectively, from 2016. Numbers of TB cases and disease incidence were highest in the WHO South-East Asia and Africa regions, and 9% of cases occurred among persons with HIV infection. Rifampicin-resistant (RR) or multidrug-resistant (MDR) (resistance to at least both isoniazid and rifampicin) TB occurred among 3.6% and 18% of new and previously treated TB cases, respectively (5.6% among all cases). Overall progress in global TB elimination was modest in 2017, consistent with that in recent years (*1*); intensified efforts to improve TB diagnosis, treatment, and prevention are required to meet global targets for 2020–2035.

TB data are reported to WHO annually by 194 member states and are reviewed and validated in collaboration with reporting entities. For countries in which case notifications did not capture all incident cases that occurred within a year (based on a standardized checklist), special studies, including TB prevalence surveys (*5*) or inventory studies (*6*), contributed to incidence estimates. For each country, 2017 disease incidence (per 100,000 HIV-negative persons and per 100 persons with HIV infection) and confidence intervals were estimated from 1) TB prevalence surveys; 2) notifications adjusted by a standard factor to account for underreporting, overdiagnosis, and underdiagnosis; 3) national inventory studies that measure the level of underreporting of detected TB cases, combined with capture-recapture modeling (*6*); and 4) national case notification data supplemented with expert opinion about case-detection gaps. Among HIV-negative persons, TB mortality estimates were based on cause of death data from civil registration and vital statistics, mortality surveys, or the product of TB incidence and case fatality. Among persons with HIV infection, TB mortality was derived from the product of incidence among persons with HIV infection and case fatality (*1*). Data on persons receiving TB preventive treatment, reported to WHO, were compared with estimates of eligible persons.

## Global TB Disease

**FIGURE 1:**
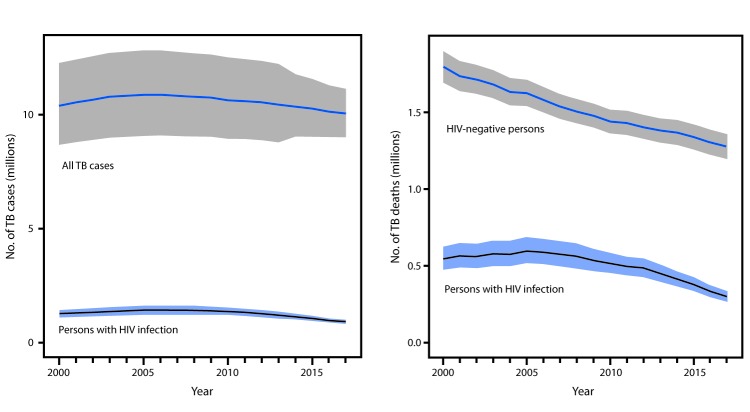
Trend in the estimated number of total tuberculosis (TB) incident cases and TB incident cases among persons with human immunodeficiency virus (HIV) infection, and trend in the estimated number of TB deaths among HIV-negative persons and persons with HIV infection, by year — worldwide, 2000–2017

In 2017, an estimated 10 million incident cases of TB occurred (133 cases per 100,000 population), a 1.8% decline from 2016 (Figure 1). Incidence has declined by an average of 1.5% per year since 2000. Estimated TB deaths declined 3.9%, from 1.64 million in 2016 to 1.57 million in 2017 (case fatality = 15.7%; 0.5% decline from 2016) (Figure 1). Among persons with HIV infection, an estimated 920,000 incident TB cases occurred in 2017, accounting for 9% of TB cases. Among this group, the estimated annual TB incidences in 2000, 2016, and 2017 were 4.5%, 2.6%, and 2.4%, respectively; in 2017, an estimated 300,000 TB deaths among persons with HIV infection occurred (case fatality = 32.6%). Overall, an estimated 558,000 incident cases of RR or MDR TB occurred in 2017, representing 5.6% of all TB cases, 3.6% of newly diagnosed TB cases, and 18% of previously treated cases. An estimated 230,000 persons died of either RR or MDR TB (case fatality = 41%).

## Regional Epidemiology of TB

**FIGURE 2:**
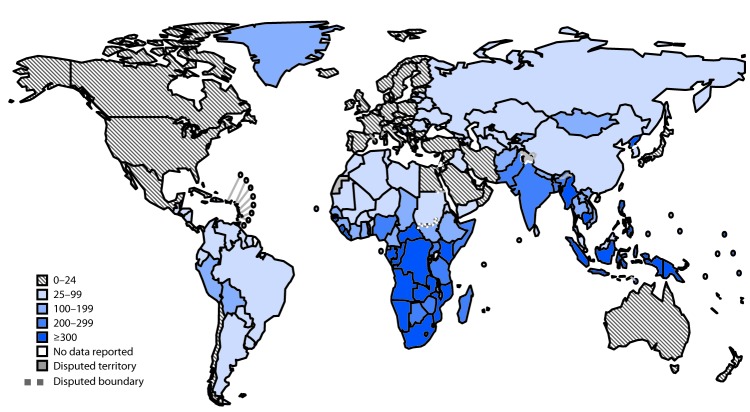
Annual tuberculosis incidence (per 100,000 population), by region — worldwide, 2017

The WHO regions of South-East Asia and Africa accounted for nearly 70% of overall global TB. Although total case numbers were higher in South-East Asia, overall incidence was similar in both regions (226 per 100,000 [South-East Asia], 237 [Africa]) (Table). Most high-incidence countries in 2017 were located in these two regions (Figure 2); however, the proportion of TB cases among persons with HIV infection in Africa (27%) was higher than that in South-East Asia (3%). Although the overall incidence of TB in the WHO European region was relatively low, the proportion of TB cases with RR or MDR TB in this region (40%) was substantially higher than that in all other regions (range = 3.6%–6.3%).

**TABLE:** Estimated number of incident tuberculosis (TB) cases, incidence, and percentage of deaths among all TB cases, TB cases among persons with human immunodeficiency virus (HIV) infection, and rifampicin-resistant (RR) or multidrug-resistant (MDR) TB cases, by World Health Organization (WHO) region — 2017

WHO region	All TB cases			TB cases among persons with HIV infection			RR or MDR TB cases		
	No. (x1,000)	Incidence*	Deaths, no. (x1,000) (fatality^§^)	No. (x1,000)	Incidence^†^	Deaths, no. (x1,000) (fatality^§^)	No. (x1,000)	Incidence*	% RR or MDR among all TB cases
**Global (all regions)**	**10,000**	**133**	**1,570 (15.7)**	**920**	**2.4**	**300 (32.6)**	**558**	**7.4**	**5.6**
African	2,480	237	665 (26.8)	663	2.5	252 (38.0)	90	8.6	3.6
Americas	282	28	24 (8.5)	30	0.87	6 (20)	11	1.1	3.9
Eastern Mediterranean	771	113	92 (11.9)	9.8	2.5	3 (30.6)	41	6.0	5.3
European	273	30	29 (10.6)	33	1.4	5 (15.2)	109	12.0	40.0
South-East Asia	4,440	226	666 (15.0)	152	4.2	28 (18.4)	192	9.7	4.3
Western Pacific	1,800	94	97 (5.4)	31	2	5 (16.1)	114	6.0	6.3

* Cases per 100,000 population.

^†^ Cases per 100 persons with HIV infection.

^§^ Per 100 TB cases.

## Use of TB Preventive Treatment

TB preventive treatment (TPT) (most commonly daily isoniazid for ≥6 months) has been found to prevent TB disease among persons who might be infected with TB and are at risk for progressing to TB disease. Current recommendations include providing TPT to persons with HIV infection (in which isoniazid has been shown to result in a 37% reduction in all-cause mortality) (*7*) and to all household contacts of patients with bacteriologically confirmed pulmonary TB disease (previously recommended only for children aged <5 years) (*7*,*8*).* In 2017, 67 and 138 countries reported data on use of TPT among eligible persons with HIV infection and children aged <5 years, respectively. Among these countries, approximately 960,000 persons with HIV received TPT (estimated coverage = 36%), similar to the number reported in 2014 (930,000). Approximately 292,000 eligible children aged <5 years received TPT in 2017, representing 23% of the estimated number of children in this group eligible for TPT.

## Discussion

In 2017, estimated TB incidence and the total number of TB deaths declined slightly worldwide; however, WHO estimates indicate that the rates of these declines are not sufficient to meet 2020 milestones (*1*). Substantial annual reductions in TB incidence and the number of TB deaths will be necessary to meet the U.N. Sustainable Development Goals and WHO End TB Strategy targets for 2030 and 2035.

The epidemiology of TB varies geographically by WHO region. In Africa, which has the highest regional prevalence of HIV infection, coinfection with HIV is a significant factor in the TB epidemic and associated mortality, and TB case fatality is highest in this region. In South-East Asia, TB incidence is similar to that in Africa; however, low HIV infection prevalence suggests that other factors, such as undernutrition or poverty, might be driving the epidemic in this region. RR or MDR TB strains present challenges in treating TB (*9*). RR or MDR TB is a major problem in Europe, where the proportion of overall cases that are RR or MDR TB is five to 10 times higher than that in all other regions. The heterogeneous regional epidemiology of TB indicates that enhanced elimination strategies based on region-specific risk factors (e.g., screening for TB among persons with HIV infection and groups at high risk, addressing poverty and malnutrition, and testing for and treating drug-resistant TB) are needed.

Successful treatment of persons with RR or MDR TB disease remains challenging, as evidenced by the high case fatality rate among RR or MDR TB patients. Two new oral treatments, delamanid and bedaquiline, have demonstrated favorable efficacy and safety profiles for treating drug-resistant TB strains (*9*). The End TB Strategy recommends ≥90% treatment coverage with new TB drugs by 2025 (*4*), thereby supporting increased use of delamanid and bedaquiline.

An estimated one quarter of the world’s population has latent TB infection and is at risk for future TB disease (*10*), which has potential to be averted by TPT (*7*,*8*). Available data indicate relatively slow uptake of TPT and a stagnation in TPT administration among persons with HIV infection in recent years (*1*); current TPT coverage falls well below the End TB Strategy target level of ≥90% coverage by 2025 (*4*). In addition to the traditional TPT regimen of ≥6 months of daily isoniazid, the recently released WHO guidelines on latent TB infection treatment support a once weekly isoniazid-rifapentine (3HP) regimen for 3 months for both adults and children (*8*). Although the rifapentine component of 3HP is substantially more expensive than conventional isoniazid regimens, the ease of use, improved adherence rates, and comparable safety and efficacy of 3HP have the potential to increase TPT coverage.

The findings in this report are subject to at least two limitations. First underlying data quality, particularly for surveillance, might affect the accuracy of estimates. Second, the differing methodologies used to generate country-level estimates might affect the comparability of estimates between regions and countries.

Current epidemiologic estimates demonstrate only modest progress in eliminating TB, as measured by incident disease, mortality, and drug resistance, and the rates of decline in these measures must increase if initial 2020 targets are to be met (*1*). Intensified efforts to improve TB diagnosis, treatment, and prevention are required to meet global targets for 2020–2035. Innovative approaches to case finding, scale-up of TPT, especially among populations at high risk, use of newer TB treatment regimens, prevention and control of HIV infection, as well as interventions tailored to specific epidemiologic contexts, will contribute to decreasing TB.

**Summary**

**What is already known about this topic?**

Worldwide, tuberculosis (TB) is the leading cause of death from a single infectious disease agent and the leading cause of death among persons living with human immunodeficiency virus (HIV) infection.

**What is added by this report?**

In 2017, an estimated 10 million incident TB cases and 1.6 million TB deaths occurred, representing reductions of 1.8% and 3.9% from 2016, respectively. TB epidemiology varied by World Health Organization region.

**What are the implications for public health practice?**

Innovative approaches to case finding, scale-up of TB preventive treatment, use of newer TB treatment regimens, and prevention and control of HIV will contribute to decreasing TB.

## Footnotes

* Latent TB infection testing by tuberculin skin test or interferon *γ*-release assay is not a requirement for initiating preventive treatment in persons with HIV infection or in household contacts aged <5 years. Persons should be screened to rule-out active TB disease before TB preventive treatment initiation.
